# The association between a body shape index and cardiovascular risk in overweight and obese children and adolescents

**DOI:** 10.1371/journal.pone.0190426

**Published:** 2018-01-03

**Authors:** Chiara Mameli, Nir Y. Krakauer, Jesse C. Krakauer, Alessandra Bosetti, Chiara Matilde Ferrari, Norma Moiana, Laura Schneider, Barbara Borsani, Teresa Genoni, Gianvincenzo Zuccotti

**Affiliations:** 1 Department of Pediatrics, V. Buzzi Children’s Hospital, University of Milan, Milan, Italy; 2 Department of Civil Engineering, The City College of New York, New York, NY, United States of America; 3 Metro Detroit Diabetes and Endocrinology, Southfield, MI, United States of America; University of Oslo, NORWAY

## Abstract

A Body Shape Index (ABSI) and normalized hip circumference (Hip Index, HI) have been recently shown to be strong risk factors for mortality and for cardiovascular disease in adults. We conducted an observational cross-sectional study to evaluate the relationship between ABSI, HI and cardiometabolic risk factors and obesity-related comorbidities in overweight and obese children and adolescents aged 2–18 years. We performed multivariate linear and logistic regression analyses with BMI, ABSI, and HI age and sex normalized z scores as predictors to examine the association with cardiometabolic risk markers (systolic and diastolic blood pressure, fasting glucose and insulin, total cholesterol and its components, transaminases, fat mass % detected by bioelectrical impedance analysis) and obesity-related conditions (including hepatic steatosis and metabolic syndrome). We recruited 217 patients (114 males), mean age 11.3 years. Multivariate linear regression showed a significant association of ABSI z score with 10 out of 15 risk markers expressed as continuous variables, while BMI z score showed a significant correlation with 9 and HI only with 1. In multivariate logistic regression to predict occurrence of obesity-related conditions and above-threshold values of risk factors, BMI z score was significantly correlated to 7 out of 12, ABSI to 5, and HI to 1. Overall, ABSI is an independent anthropometric index that was significantly associated with cardiometabolic risk markers in a pediatric population affected by overweight and obesity.

## Introduction

Obesity is one of the most serious international health concerns [1,2]. The increased prevalence of obesity in children and adolescents has led to heightened awareness about its impacts on cardiovascular and metabolic health.

Obesity has been shown to be an independent predictor of some adverse cardiovascular events, increasing the mortality rate when compared to normal weight subjects [3,4]. In children and adolescents obesity is associated with cardiometabolic risk factors, including hypertension, hyperlipidemia, insulin resistance and type 2 diabetes, which can lead to cardiometabolic diseases during adulthood [5,6]. This association implies the need to identify easy, low-cost, and reliable methods to identify those at most risk.

Since the 1970s, the classification of obesity, for both adults and children, has been based on weight/height^2^ (BMI) [2]. BMI identifies patients who are at increased risk of cardiometabolic risk factors [7]. BMI was initially applied to adults, and shown to be highly correlated with adiposity and cardiovascular risk [8]. In children, BMI z scores are usually used instead of BMI as standardized measures of weight adjusted for child age and sex. However, BMI is only a surrogate measure of body fat, because it measures excess weight rather than excess fat, and the association of childhood BMI z score with cardiometabolic risk is nonlinear [9].

Given these limitations and the heightened awareness of the importance of the sites of fat deposition for the pathophysiology of cardiometabolic disturbances, researchers have used other body measures such waist circumference (WC), waist-to-height ratio, and waist-to-hip ratio to further specify risk, primarily in adult populations. The utility of these anthropometric indexes in children and adolescents as markers of metabolic comorbidities remains unclear [10–17].

Recently, new expressions for normalized WC (A Body Shape Index, ABSI) and hip circumference (Hip Index, HI) have been introduced and have been shown to be risk factors for mortality and for cardiovascular disease independent of BMI and of each other [17,18]. Considering that previous studies have shown that abdominal fat is associated with cardiovascular risk, even in children and adolescents, an index like ABSI, which expresses WC relative to height and weight, can potentially be a complementary predictor of cardiovascular risk alongside BMI [19–21]. A study of an adult population recently showed that ABSI was associated with several components of the metabolic syndrome [22].

To our knowledge, no data are available about the relationship between ABSI and cardiovascular risk factors in the pediatric population affected by overweight and obesity.

The main objective of this study is to find the association between the new anthropometric indexes ABSI and HI and cardiometabolic risk factors and the presence of obesity-related comorbidities in overweight and obese children and adolescents.

## Methods

We performed an observational cross-sectional study. The study participants were recruited from the obesity outpatient clinic of V. Buzzi Children's Hospital in Milan (Italy) by retrieving information from the medical records of the patients from April 1^st^ 2010 to April 30^th^ 2017. Inclusion criteria include age from 2 to 18 years and diagnosis of overweight or obesity as detailed below. We excluded children and adolescents affected by genetic/syndromic obesity, familial hyperlipidemia, use of antihypertensive, antidiabetic, or lipid-lowering medication or medications such as corticosteroids that affect body composition.

When referred to the clinic, every patient was evaluated by the medical team, including measurement of systolic (SBP) and diastolic blood pressure (DPB) and pubertal evaluation. Pubertal stage was assessed according to Tanner [23]. Tanner stages 1 and 2 were combined as prepubertal/early pubertal, stages 3 and 4 as pubertal, and stage 5 as postpubertal development. Every patient was also evaluated by a trained dietician, who performed anthropometric measurements (including waist and hip circumference) and bioelectrical impedance analysis (BIA) as described below.

Every patient was invited to perform blood tests described in the section below within 7 days from the medical visit. Moreover, patients were also invited to be screened within 15 days from the medical evaluation by liver ultrasound for detection and quantification of hepatic steatosis (detailed description below).

All procedures were between 8.00–10.00 AM and were paid for by the National Health System, in accordance with institutional guidelines.

All patients or their parents gave written consent to participate in the study. The study was approved by the Ethics Committee of ASST-FBF-Sacco (Milan, Italy) and conducted in accordance with the Declaration of Helsinki.

### Blood pressure

Peripheral brachial BP was measured by a trained observer using an aneroid sphygmomanometer (TEMA, Model Certus, Italy) 3 times after the patient sat still for at least 5 minutes. Hypertension was defined as BP ≥95^th^ percentile for age, sex, and height as elsewhere reported [24].

### Anthropometric measurements

Weight was measured using a medical-certified scale (SECA, Hamburg, Deutschland). Height was measured using a children's medical-certified stadiometer (SECA, Hamburg, Deutschland). BMI was calculated as body mass (W, kg) divided by height (H, m) squared. The BMI values were transformed into BMI z scores using WHO reference values for pediatric BMI [25].

Overweight was defined by BMI z score between 1 and 2, and obesity as BMI z score ≥ 2 (i.e., at least 2 standard deviations above the age- and sex-specific expected value).

Waist and hip circumferences (WC, HC) were measured in centimeters to the nearest 0.1 cm twice using inextensible anthropometric tape positioned parallel to the floor. WC was measured midway between the lowest border of rib cage and the upper border of iliac crest, at the end of normal expiration. HC was measured at the widest part of the hip at the level of the greater trochanter [26].

ABSI was calculated using the following formula ABSI = WC(m)/[BMI^2/3^ × H (m)^1/2^] [17].

HI was calculated using the formula HI = HC(cm) * (H / 166 cm)^0.310^ * (W / 73 kg)^-0.482^ [19]. ABSI and HI values were converted to age and sex specific z scores calculated based on USA population normals [18].

### Blood tests

Blood samples were obtained after a 10-hour overnight fast for measurement of plasma glucose, insulin, alanine aminotransferase (ALT), aspartate aminotransferase (AST), total cholesterol (TC), high-density lipoprotein cholesterol (HDL), low-density lipoprotein cholesterol (LDL), and triglyceride levels (TG) using standard laboratory methods. All samples were analyzed by the same laboratory. Blood was drawn for glycated haemoglobin (HbA1c) analysis using a fully automated high-performance liquid chromatography system (Variant II, Bio-Rad Laboratories, Munich, Germany).

The homeostasis model assessment of insulin resistance (HOMA-IR) was calculated using the following formula: fasting plasma insulin [in μIU/ml] + fasting plasma glucose [in mmol/l] ÷ 22.5 [27]. The threshold used for insulin resistance was a HOMA-IR value ≥95^th^ percentile adjusted for gender and Tanner stages according to reference value of HOMA-IR in the Italian population as described elsewhere [28]. Pancreatic β-cell function (HOMA- β %) was measured as (20 x fasting insulin in mU/l)/(fasting glucose in mmol/l– 3.5) [27]. Reduced β-cell function was defined as HOMA- β % value ≤5^th^ percentile, adjusted for gender and Tanner stage, according to reference values of HOMA- β % in the Italian population as described elsewhere [28].

We used standard cut-off values to define high-risk levels of total cholesterol (hypercholesterolemia ≥200 mg/dl), HDL cholesterol (HypoHDL <40 mg/dl, <50 mg/dl for females >16 years), LDL cholesterol (HyperLDL ≥130 mg per deciliter), triglycerides (HyperTG, ≥100 mg/dl for children aged 0–9 years and ≥ 130 mg/dl for those aged 10–19 years) [29], and triglycerides/HDL cholesterol ratio (TG-HDL, >2.2) [30,31]. Impaired fasting glucose (IFG) was defined as fasting plasma glucose level above 100 mg/dl according to the American Diabetes Association criteria [32].

### Liver ultrasonography

Steatosis was identified by ultrasonography based on echogenicity and visualization of vasculature, parenchyma and diaphragm. Steatosis was scored as follows: absent (score 0), defined as normal liver echotexture; mild (score 1), slight and diffuse increase in fine parenchymal echoes with normal visualization of diaphragm and portal vein borders; moderate (score 2), moderate and diffuse increase in fine echoes with slightly impaired visualization of portal vein borders and diaphragm; severe (score 3), fine echoes with poor or no visualization of portal vein borders, diaphragm, and posterior portion of the right lobe [33]. All examinations were performed using an Acuson S2000 ultrasound system (Siemens, Germany) with linear and convex transducers (frequency bandwidth 4-14MHz) by the same operator, who had 10 years of experience in ultrasonography studies.

### Bioelectric impedance analysis

Body fat mass expressed in kg (FM kg) and percent body fat mass (FM %) was determined by a body fat analyzer (Tanita, 780MA, Tanita Corp of America, Inc, Arlington Heights, Ill). This method for estimating percentage of body fat has a high correlation with dual-energy x-ray absorptiometry in children [34].

### Metabolic syndrome

For metabolic syndrome (MS), we used the International Diabetes Federation (IDF) definitions, which are as follows. For children aged from 10 to 16 years, metabolic syndrome is diagnosed with WC above either the 90^th^ percentile or the adult cut-off and the presence of two or more other features (TG ≥150 mg/dl, HDL-cholesterol < 40 mg/dl, SBP ≥ 135 mg/dl, DBP ≥85 mg/dl, or fasting glucose > 100 mg/dl). For patients age ≥ 16 years, MS is diagnosed with WC ≥ 94 cm for men and ≥ 80 cm for women, plus any two of the following factors: TG ≥150 mg/dl, HDL-cholesterol < 40 mg/dl in males and < 50 mg/dl in females, SBP ≥ 135 mmHg, DBP ≥85 mmHg, or fasting glucose > 100 mg/dl [35].

### Statistical analysis

Multivariate linear regression with age, sex (entered as 0 = male, 1 = female), and z scores of height, BMI, ABSI, and HI as predictors was used to evaluate which demographic and anthropometric factors were associated with each cardiometabolic risk factor, entered as a continuous predicand variable. Regression coefficients were standardized to enable easier comparability across predicands, through dividing each predicand by its standard deviation.

Additionally, multivariate logistic regression with the same predictors was used to evaluate which factors were associated with above-threshold value of each risk factor (including above-threshold values for the components of metabolic syndrome; presence of metabolic syndrome for those aged at least 10 years; HOMA-IR ≥95^th^ percentile; HOMA-β % ≤5^th^ percentile; and presence of hepatic steatosis), entered as a dichotomous predicand variable (0 = absent, 1 = present). All analysis was carried out in the R computing environment.

## Results

From April 1^st^ 2010 to April 30^th^ 2017 we recruited 217 patients (114 males, 103 female, mean age 11.3 years ± 2.9 standard deviation [SD]) who fulfilled the inclusion criteria. Table 1 gives the characteristics of the patients.

**Table 1:** Patient characteristics.

Patients’ characteristics	N (%)
**Gender**	
**Female**	103 (47)
**Male**	114 (53)
**Ethnicity**	
**Caucasian**	179 (82)
**Hispanic**	15 (7)
**Asian**	15 (7)
**African**	8 (4)
**Age (Years) (Mean, SD)**	11.3 (2.9)
**Age 3–5 years**	10 (5)
**Age 6–9 years**	63 (29)
**Age 10–17 years**	138 (65)
**Tanner stage**	
**Prepubertal/early pubertal**	7(35)
**Pubertal**	94 (44)
**Post pubertal**	47 (22)
**Weight (kg) (mean, SD)**	63 (20)
**Height (cm) (mean, SD)**	150 (16)
**Height z score (mean, SD)**	0.70 (1.11)
**BMI (mean, SD)**	27.3 (4.4)
**BMI z score (mean, SD)**	2.81 (0.91)
**Overweight**	29 (13)
**Obese**	188 (87)
**ABSI z score (mean, SD)**	-0.75 (1.05)
**HI z score (mean, SD)**	0.06 (1.36)

ABSI: A Body Shape Index; BMI: body mass index, HC: hip circumference, HI: Hip Index; N: number; SD: standard deviation

138 patients (65%) were older than 10 years. 35% were prepubertal and 22% post-pubertal. 188 patients (87%) were obese. Mean ABSI z score was -0.75 ± 1.05 SD. Mean HI z score was 0.06 ± 1.36 SD. All patients were non-smokers.

Table 2 shows means and standard deviations of blood pressure, biochemical variables and fat mass in our patients.

**Table 2:** Blood pressure, biochemical variables and fat mass.

Variable	Total		Males		Females	
	N	Mean ± SD	N	Mean ± SD	N	Mean ± SD
**SBP (mmHg)**	205	107 (14)	111	107 (13)	94	107 (15)
**DBP (mmHg)**	205	65 (10)	111	65 (9)	94	64 (10)
**Glucose (mg/dl)**	207	87 (10)	108	87 (10)	99	87 (11)
**Insulin (μU/ml)**	189	12.3 (7.5)	101	12.0 (8.1)	88	12.6 (6.8)
**Hba1c (mmol/mol)**	189	35.9 (4.4)	98	35.9 (4.0)	91	35.9 (4.8)
**HOMA IR**	184	2.66 (1.74)	98	2.56 (1.89)	86	2.78 (1.55)
**HOMA β%**	184	199 (138)	98	189 (150)	86	211 (124)
**TC (mg/dl)**	213	162 (29)	113	162 (29)	100	162 (28)
**HDL (mg/dl)**	211	48 (10)	112	46 (10)	99	49 (10)
**LDL(mg/dl)**	211	96 (25)	112	97 (26)	99	96 (24)
**TG (mg/dl)**	216	89 (51)	114	93 (59)	102	85 (40)
**TG/HDL**	211	2.04 (1.50)	112	2.22 (1.78)	99	1.82 (1.06)
**AST (U/L)**	197	26.2 (8.8)	104	28.4 (9.3)	93	23.8 (7.5)
**ALT (U/L)**	195	26.6 (19.0)	103	30.6 (22.9)	92	22.1 (12.1)
**FM (kg)**	129	23.7 (9.6)	73	22.0 (7.3)	56	25.9 (11.7)
**FM (%)**	129	34.7 (6.0)	73	33.6 (5.9)	56	36.2 (5.8)

ALT: alanine aminotransferase, AST: aspartate aminotransferase, DPB: diastolic blood pressure, FM: fat mass, HbA1c: glycated hemoglobin; HDL: high-density lipoprotein cholesterol, HOMA–IR: Homeostatic Model Of Assessment–IR, HOMA β%: Homeostatic Model Of Assessment β%, LDL: low-density lipoprotein cholesterol, N: number of subjects with available data, SBP: systolic blood pressure, SD: standard deviation, TC: total cholesterol, TG: triglycerides; U: Unit.

Table 3 shows the cardiometabolic comorbidities of our patients.

**Table 3:** Prevalence of cardiometabolic risk factors and obesity-related comorbidities.

Cardiometabolic risk factors	N^‡^/total population	Female N^‡^/total F	Males N^‡^/total M	Age 3–5 y (N^‡^/tot)	Age 6–9 y (N^‡^/tot)	Age 10–17 y (N^‡^/tot)	Pre pubertal (N^‡^/tot)	Pubertal (N^‡^/tot)	Post pubertal (N^‡^/tot)
**Systolic** hypertension*	16/205	7/94	9/111	0/10	0/61	16/134	0/72	10/88	6/44
**Diastolic** hypertension*	7/205	3/94	4/111	0/10	0/61	7/134	0/72	5/88	2/44
**HyperTC**	22/213	10/100	12/113	0/10	5/63	17/140	6/75	10/91	6/46
**Hyper LDL**	25/211	8/99	17/112	0/10	8/62	17/139	10/74	11/91	4/45
**HypoHDL**	46/209	19/99	27/112	5/10	13/62	28/139	20/74	18/91	8/45
**HyperTG**	44/216	17/102	27/114	2/10	19/63	23/143	21/75	17/93	6/47
**TG/HDL≥2.2**	72/211	28/99	44/112	3/10	23/62	46/139	32/74	29/91	11/45
**IFG**	17/207	7/99	10/108	0/9	5/59	12/139	4/71	9/91	4/44
**HOMA-IR ≥95**^**th**^ **centile** ^†^	46/184	13/86	33/98	2/7	11/52	33/125	17/61	22/85	7/37
**HOMA β cell ≤5**^**th**^ **centile**^†^	10/184	3/86	7/98	0/7	4/52	6/125	5/61	5/85	0/37

* for gender, age and height

^†^ for gender, and Tanner stages.

N^‡^: number of subjects affected.

N^‡^/tot: the number of subjects affected and the total number assessed in each subgroup.

DBP ≥ 95^th^ percentile; F: female; Hyper LDL ≥130 mg/dl; HyperTC ≥200 mg/dl; HyperTG ≥150 mg per deciliter HypoHDL <40 mg/dl (<50 for females aged ≥ 16 y); IFG: impaired fasting glucose; M: male; SBP ≥ 95^th^ percentile; y: years.

As defined based on age, height and gender specific percentiles, 16/205 (7.8%) patients had systolic hypertension and 3.4% diastolic hypertension All the hypertensive patients were aged 10–17 years old. Detected alterations in lipid profile included hypoHDL cholesterol values (22%), hyperTG (20%), hypercholesterolemia (10.3%), and hyperLDL cholesterol (10%). TG/HDL was ≥2.2 in 34% of patients.

IFG was found in 8.2% of our patients (17/207). HOMA-IR value ≥95^th^ was found in 25% of our population (46/184), with a higher frequency in males compared to females (33.6% versus 15.1%). Reduced β cell function was found in 5.4% of patients (10/184), with an incidence of 7.1% in males compared to 3.4% in females.

Relatively few patients met the MS IDF definition (10/132 out of those ≥ 10 years of age), even though most (68/132) had WC above 90^th^ percentile.

The prevalence of hepatic steatosis was 30% (50/162 patients); of those patients 6% had severe steatosis.

Multivariate linear regression (Table 4) showed that ABSI z-score correlated with 10 out of 15 risk markers expressed as continuous variables, while BMI z score showed a significant correlation with 9 and HI only with 1.

ABSI z score showed a significant positive correlation with serum insulin (standardized regression coefficient 0.22), HOMA–IR (0.18), HOMA β% (0.17), TC (0.15), LDL (0.20), TG (0.15), ALT (0.19), FM% (0.17), TG/HDL (0.21). BMI z score positively correlated with SBP (standardized regression coefficient 0.56), DBP (0.37), insulin (0.28), HOMA β% (0.29), TG (0.24), ALT (0.35), FM % (0.98), TG/HDL (0.29). Both BMI z score and ABSI z score showed a significant negative correlation with HDL cholesterol (coefficient -0.34 and -0.25 respectively). HI was associated only with FM% (coefficient 0.15) (Table 4).

**Table 4:** Standardized coefficients (with 95% confidence intervals) from multiple linear regression of risk factors on sex, age, and anthropometric indices.

	N	Sex (M = 0, F = 1)	Age (y)	Height z score	BMI z score	ABSi z score	HI z score	Regression adjusted R^2^
**SBP**	187	0.19 (-0.06, 0.43)	**0.28 (0.23, 0.33)**	**0.15 (0.04, 0.26)**	**0.56 (0.40, 0.71)**	0.07 (-0.04, 0.19)	-0.05 (-0.13, 0.03)	0.47
**DBP**	187	0.04 (-0.24, 0.33)	**0.21 (0.16, 0.27)**	0.09 (-0.03, 0.22)	**0.37 (0.19, 0.55)**	-0.01 (-0.14, 0.12)	0.01 (-0.09, 0.10)	0.27
**Glucose**	189	0.08 (-0.25, 0.41)	-0.01 (-0.08, 0.05)	-0.05 (-0.20, 0.10)	-0.02 (-0.23, 0.20)	0.12 (-0.03, 0.27)	-0.04 (-0.15, 0.07)	-0.01
**Insulin**	174	**0.44 (0.15, 0.74)**	**0.15 (0.09, 0.21)**	**0.21 (0.08, 0.35)**	**0.28 (0.08, 0.47)**	**0.22 (0.09, 0.36)**	0.01 (-0.09, 0.10)	0.14
**Hba1c**	172	-0.06 (-0.39, 0.26)	**0.10 (0.04, 0.16)**	-0.01 (-0.16, 0.14)	0.17 (-0.04, 0.38)	-0.02 (-0.18, 0.13)	-0.10 (-0.22, 0.02)	0.07
**HOMA IR**	199	**0.36 (0.06, 0.67)**	**0.11 (0.05, 0.17)**	**0.19 (0.05, 0.32)**	0.15 (-0.04, 0.35)	**0.18 (0.03, 0.32)**	-0.01 (-0.11, 0.10)	0.07
**HOMA β**	169	**0.43 (0.12, 0.73)**	**0.16 (0.10, 0.22)**	**0.25 (0.11, 0.40)**	**0.29 (0.09, 0.49)**	**0.17 (0.03, 0.31)**	0.01 (-0.09, 0.12)	0.14
**TC**	195	0.07 (-0.24, 0.39)	0.01 (-0.04, 0.08)	-0.04 (-0.18, 0.10)	0.05 (-0.15, 0.25)	**0.15 (0.01, 0.30)**	0.02 (-0.09, 0.12)	-0.01
**HDL**	193	0.03 (-0.28, 0.33)	-0.04 (-0.10, 0.02)	-0.04 (-0.17, 0.10)	**-0.34 (-0.53, -0.14)**	**-0.25 (-0.39, -0.11)**	0.02 (-0.08, 0.13)	0.10
**LDL**	193	0.07 (-0.24, 0.38)	0.02 (-0.03, 0.08)	-0.02 (-0.15, 0.12)	0.09 (-0.10, 0.29)	**0.20 (0.06, 0.35)**	0.03 (-0.07, 0.14)	0.02
**TG**	198	0.04 (-0.27, 0.35)	0.04 (-0.02, 0.10)	-0.03 (-0.17, 0.10)	**0.24 (0.04, 0.44)**	**0.15 (0.01, 0.30)**	-0.07 (-0.18, 0.03)	0.03
**TG/HDL**	193	-0.02 (-0.32, 0.29)	0.03 (-0.03, 0.09)	-0.05 (-0.18, 0.09)	**0.29 (0.09, 0.49)**	**0.21 (0.06, 0.35)**	-0.05 (-0.15, 0.05)	0.06
**AST**	181	-**0.46 (-0.76, -0.16)**	-0.06 (-0.12, 0.00)	0.05 (-0.09, 0.18)	0.16 (-0.04, 0.35)	0.13 (-0.02, 0.29)	-0.07 (-0.17, 0.04)	0.15
**ALT**	180	-0.29 (-0.58, 0.01)	**0.07 (0.01, 0.13)**	**0.17 (0.04, 0.30)**	**0.35 (0.16, 0.54)**	**0.19 (0.05, 0.33)**	0.01 (-0.09, 0.11)	0.17
**FM %**	114	**0.77 (0.45, 1.08)**	**0.07 (0.00, 0.14)**	-0.09 (-0.23, 0.05)	**0.98 (0.77, 1.18)**	**0.17 (0.00, 0.33)**	**0.15 (0.01, 0.29)**	0.47
**Number of significant associations**		5/15	8/15	5/15	9/15	10/15	1/15	

Coefficients represent standard deviations of risk factor (see Table 1 for the standard deviation values) per year of age, female vs. male sex, or unit change in z score. Values in bold represent significant associations.

ABSI: A Body Shape Index; ALT: alanine aminotransferase, AST: aspartate aminotransferase, BMI: Body Mass Index, DPB: diastolic blood pressure, F: females, FM: fat mass, HbA1c: glycated gemoglobin, HDL: high-density lipoprotein cholesterol, HI: Hip Index, HOMA–IR: Homeostatic Model Of Assessment–IR, HOMA β%: Homeostatic Model Of Assessment β%, LDL: low-density lipoprotein cholesterol, M: males, N: number of subjects, SBP: systolic blood pressure, TC: total cholesterol, TG: triglycerides, y: years

Table 5 shows odds ratios from multiple logistic regression of dichotomous risk factors on sex, age, and anthropometric indices.

**Table 5:** Odds ratios (with 95% confidence intervals) from multiple logistic regression of dichotomous risk factors on sex, age, and anthropometric indices.

	N‡/tot	Sex (M = 0, F = 1)	Age (y)	Height z score	BMI z score	ABSI z score	HI z score	Regression pseudo R^2^
High SBP*	16/187	0.8 (0.2, 3.2)	**1.7** **(1.3, 2.3)**	1.0 (0.5, 1.8)	**3.5** **(1.6, 8.7)**	0.7 (0.4, 1.4)	**0.7** **(0.5, 1.0)**	0.32
High DBP*	7/187	0.6 (0.1, 3.9)	1.4 (1.0, 2.0)	0.7 (0.3, 1.6)	**2.4** **(1.0, 6.2)**	0.9 (0.3, 2.1)	0.8 (0.5, 1.5)	0.22
**HyperTC**	20/195	1.3 (0.5, 3.9)	1.2 (1.0, 1.5)	1.2 (0.8, 1.9)	1.5 (0.8, 2.6)	1.3 (0.8, 2.2)	1.0 (0.7, 1.5)	0.17
**HyperTG**	38/198	1.4 (0.6, 3.2)	1.1 (0.9, 1.2)	1.2 (0.8, 1.7)	1.6 (1.0, 2.6)	**1.6** **(1.1, 2.4)**	0.8 (0.6, 1.1)	0.25
**HyperLDL**	22/193	0.6 (0.2, 1.5)	1.1 (0.9, 1.3)	1.1 (0.7, 1.7)	1.4 (0.7, 2.4)	1.0 (0.6, 1.5)	1.1 (0.8, 1.5)	0.19
**HypoHDL**	41/193	1.2 (0.5, 2.6)	1.0 (0.9, 1.2)	0.8 (0.6, 1.2)	**1.8** **(1.1, 3.0)**	**1.5** **(1.0, 2.2)**	1.1 (0.9, 1.5)	0.24
**TG/HDL ≥2.2**	66/193	1.0 (0.5, 2.0)	1.1 (1.0, 1.3)	1.2 (0.9, 1.6)	**1.7** **(1.1, 2.8)**	**1.4** **(1.0, 2.0)**	0.8 (0.7, 1.1)	0.23
**IFG**	16/189	0.8 (0.2, 2.4)	1.0 (0.8, 1.3)	0.9 (0.5, 1.4)	1.1 (0.5, 2.2)	1.0 (0.6, 1.7)	**0.7** **(0.5, 1.0)**	0.14
**MS** ^†^	12/126	1.4 (0.3, 6.0)	1.1 (0.7, 1.7)	1.1 (0.6, 2.0)	**4.7** **(1.7, 18.7)**	1.3 (0.6, 2.6)	0.8 (0.5, 1.4)	0.20
**HOMA-IR ≥95**^**th**^ **centile** ^‡^	41/169	0.6 (0.2, 1.3)	**1.3** **(1.1, 1.5)**	**1.8** **(1.2, 2.7)**	1.7 (1.0, 3.0)	**1.7** **(1.1, 2.5)**	1.0 (0.7, 1.3)	0.34
**HOMA- β ≤ 5**^**th**^ **centile** ^‡^	10/169	**0.1** **(0.0, 0.6)**	**0.5** **(0.3, 0.8)**	0.7 (0.3, 1.4)	**0.2** **(0.0, 0.6)**	0.5 (0.2, 1.1)	1.1 (0.7, 1.8)	0.24
**Hepatic steatosis**	46/147	0.7 (0.3, 1.8)	**1.3** **(1.1, 1.5)**	1.1 (0.7, 1.6)	**3.6** **(1.9, 7.5)**	**1.7** **(1.1, 2.7)**	0.9 (0.7, 1.3)	0.35
**Number of significant associations**		1/12	4/12	1/12	7/12	5/12	2/12	

* for gender, age and height

^†^ Patients age ≥ 10 years only

^‡^ for gender, and Tanner stages.

N^‡^/tot: the number of subjects affected out of the subgroup of patients with available parameters.

The odds ratios are per year of age, female vs. male sex, or unit change in z score. Values in bold represent significant associations.

DBP ≥ 95^th^ percentile; Hyper LDL ≥130 mg/dl; Hyper TC ≥200 mg/dl; HyperTG ≥150 mg/dl; HypoHDL <40 mg/dl (<50 for females aged ≥ 16 y); IFG: impaired fasting glucose; SBP ≥ 95^th^ percentile; TG-HDL ratio > 2.2

BMI z score was positively correlated to probability of systolic hypertension (odds ratio [OR] 3.5; Table 5), diastolic hypertension (2.4), hypoHDL (1.8), MS (4.7) and presence of hepatic steatosis (3.6) TG/HDL ≥ 2.2 (1.7). ABSI was correlated with hypoHDL (OR 1.5), hyperTG (1.6), HOMA-IR value ≥95% (1.7), TG/HDL ≥2.2 (1.4) and hepatic steatosis (1.7).

## Discussion

This is the first study that investigates the potential relationship in overweight and obese children and adolescents between the new anthropometric indexes ABSI and HI, along with BMI, and cardiovascular and obesity-related risk factors.

In multiple linear regression of cardiometabolic variables on anthropometric indexes, ABSI z score showed significant correlation with 10/15 cardiometabolic risk markers, expressed as continuous variables: positive correlation with fasting insulin, HOMA-IR and HOMA β%, TC, LDL, TG, ALT, TG/HDL as well as with FM%, and a negative correlation with HDL. BMI z score showed significant correlation with 9/15 markers: positive correlations with SBP, DBP, insulinemia, HOMA β%, TG, ALT, TG/HDL, FM%, and a negative correlation with HDL. Thus, ABSI z score was found to correlate with glucose metabolism indexes and the whole lipid profile, including TG/HDL, which was recently found as a biomarker of atherogenic dyslipidemia and altered cardiometabolic risk. BMI was positively associated with blood pressure and FM% (0.98 –C.I. 95% 0.77–1.18). Our results are intriguing considering that ABSI, which expresses WC relative to height and weight, has been proposed as a new and more effective method to quantify the specific risk associated with abdominal obesity (AO). The role of AO has been extensively studied in the last years in adult population, but in children is far from being completely elucidated [36]. Visceral fat, due to its location and metabolic characteristics, contributes to distorted metabolism to a much greater extent than subcutaneous fat [37]. Findings from a recent systematic review showed that central body fat deposition in children and adolescents seems to increase the probability of having cardio-metabolic risk factors [38]. Our results support the association between AO and worse lipid profile and glucose metabolism indexes, suggesting that AO promotes a cluster of atherogenic and diabetes-related risk factors. On the other hand, when considering the percentage of FM, BMI showed a strong association compared to ABSI and HI (regression coefficient 0.98 *vs* 0.17 *vs* 0.15, respectively), suggesting that other sites than the abdominal, including subcutaneous fat around the hip, could be over-represented in children and adolescents with obesity. Recently, ectopic fat, such as hepatic and pancreatic fat, was more fully investigated in pediatric populations, and some authors proposed that its accumulation could contribute to the risk of metabolic syndrome and impaired insulin response [39,40]. In support of this hypothesis we found that BMI showed a stronger correlation than ABSI (regression coefficient 0.35 *vs* 0.19) with ALT level, the most widely used first line marker of nonalcoholic fatty liver disease [41]. Imaging techniques would be useful in assessing this hypothesis.

We also analyzed cardiometabolic risk factors as dichotomous variables, using the most widely accepted thresholds to identify elevated cardiovascular risk. We found that ABSI was significantly correlated with 5/12 obesity-related cardiovascular risk factors: hypoHDL, HOMA-IR value ≥95^th^, hyperTG, elevated TG/HDL and the presence of hepatic steatosis. HI was significantly correlated only with impaired fasting glucose.

Using multivariate logistic regression to model the likelihood of above-threshold values, BMI was associated with a greater number of obesity-related cardiovascular risk factors (7/12) compared to ABSI, including systolic and diastolic hypertension, hypoHDL, elevated TG/HDL, metabolic syndrome, reduced β cell function and hepatic steatosis. The last two findings support the role of BMI in representing also ectopic fat accumulation (such as pancreatic and hepatic fat) in children, as discussed previously.

Our study suggests that in children and adolescents affected by overweight and obesity, ABSI seemed a more suitable cardiovascular risk predictor when considering metabolic markers, while on the other hand BMI was better correlated with the presence of obesity-related threshold-based risk factors.

We can speculate about the stronger association between ABSI and many cardiometabolic markers compared to the weaker association of ABSI with obesity-related conditions expressed in terms of thresholds. It is well known that cut points are not precise for delineating those at excess risk and limit the accurate identification of the at-risk child. Considering risk factors as continuous variables, we found that ABSI correlated with many cardiometabolic markers, further suggesting that the current cut-offs are incomplete indicators of increased risk. It is likely that in the future these thresholds will be revised or made more nuanced based on accumulating population-based findings.

Our study has some limitations. The population we analyzed was relatively small in size, constraining the power of multivariate regression to identify and rank anthropometric predictors, and limited to participants in a pediatric obesity clinic. For many of the risk factors and for the presence of MS, only a small percentage of the cohort was above the threshold, suggesting the need for a much larger sample to better quantify associations of risk factors and MS with anthropometrics in children and adolescents. With a larger sample size, it will also be possible to investigate nonlinear associations of BMI and HI with risk factors, as previously found for mortality risk [18]. Additionally, only a small percentage of patients were of non-Caucasian ethnicity. Large and long-term population studies are therefore needed to better establish the value of these new indexes in pediatric populations.

## Conclusions

The recently proposed ABSI was found to complement BMI as a marker of cardiometabolic and obesity-related risk factors in overweight and obese children attending an outpatient clinic. Overall, ABSI was of comparable power to BMI as a predictor of risk factors. ABSI seemed a more suitable cardiovascular risk predictor when considering metabolic markers, while on the other hand BMI was better correlated with the presence of obesity-related threshold-based risk factors.

## References

[1] Bray GA, Kim KK, Wilding JPH World Obesity Federation. Obesity: a chronic relapsing progressive disease process. A position statement of the World Obesity Federation. Obes Rev. 2017;18:715–723. doi: 10.1111/obr.12551 28489290 10.1111/obr.12551

[2] Mameli C, Mazzantini S, Zuccotti GV. Int J Environ Res Public Health. 2016; 13 pii: E838. doi: 10.3390/ijerph13090838 27563917 10.3390/ijerph13090838 PMC5036671

[3] Van Putte-Katier N, Rooman RP, Haas L, Verhulst SL, Desager KN, Ramet J, et al Early cardiac abnormalities in obese children: importance of obesity per se versus associated cardiovascular risk factors. Pediatr Res. 2008; 64:205–209. doi: 10.1203/PDR.0b013e318176182b 18391840 10.1203/PDR.0b013e318176182b

[4] Hubert HB, Feinleib M, McNamara PM, Castelli WP. Obesity as an independent risk factor for cardiovascular disease: a 26-year follow-up of participants in the Framingham Heart Study. Circulation. 1983;67:968–977. 6219830 10.1161/01.cir.67.5.968

[5] Friedemann C, Heneghan C, Mahtani K, Thompson M, Perera R, Ward AM. Cardiovascular disease risk in healthy children and its association with body mass index: systematic review and meta-analysis. BMJ.2012;345:e4759 doi: 10.1136/bmj.e4759 23015032 10.1136/bmj.e4759 PMC3458230

[6] Mameli C, Zuccotti GV, Carnovale C, Galli E, Nannini P, Cervia D, et al An update on the assessment and management of metabolic syndrome, a growing medical emergency in paediatric populations. Pharmacol Res. 2017;119: 99–117. doi: 10.1016/j.phrs.2017.01.017 28111263 10.1016/j.phrs.2017.01.017

[7] Weigley ES. Adolphe Quetelet. Am J Clin Nutr. 2000;71:853 (letter). 10702194 10.1093/ajcn/71.3.853

[8] Khosla T, Lowe CR. Indices of obesity derived from body weight and height. Br J Prev Soc Med. 1967;21:122–128. 6033482 10.1136/jech.21.3.122 PMC1059084

[9] Flegal KM, Ogden CL. Childhood obesity: are we all speaking the same language? Adv Nutr. 2011;2:159S–66S. doi: 10.3945/an.111.000307 22332047 10.3945/an.111.000307 PMC3065752

[10] Aristizabal JC, Barona J, Hoyos M, Ruiz M, Marín C. Association between anthropometric indices and cardiometabolic risk factors in pre-schoolchildren. BMC Pediatr. 2015. 6;15–170. 10.1186/s12887-015-0500-y PMC4636828 26546280

[11] Hashemipour M, Soghrati M, Malek Ahmadi M, Soghrati M. Anthropometric indices associated with dyslipidemia in obese children and adolescents: a retrospective study in isfahan. ARYA Atheroscler. 2011;7:31–39. 22577442 PMC3347839

[12] Maffeis C, Banzato C, Talamini G.; Obesity Study Group of the Italian Society of Pediatric Endocrinology and Diabetology. Waist-to-height ratio, a useful index to identify high metabolic risk in overweight children. J Pediatr. 2008;152:207–213. doi: 10.1016/j.jpeds.2007.09.021 18206690 10.1016/j.jpeds.2007.09.021

[13] Kelishadi R, Mirmoghtadaee P, Najafi H, Keikha M. Systematic review on the association of abdominal obesity in children and adolescents with cardio-metabolic risk factors. J Res Med Sci. 2015;20:294–307. 26109978 PMC4468236

[14] Morandi A, Miraglia Del Giudice E, Martino F, Martino E, Bozzola M, Maffeis C. Anthropometric indices are not satisfactory predictors of metabolic comorbidities in obese children and adolescents. J Pediatr. 2014;165:1178–1183. doi: 10.1016/j.jpeds.2014.07.004 25112691 10.1016/j.jpeds.2014.07.004

[15] Garnett SP, Baur LA, Srinivasan S, Lee JW, Cowell CT. Body mass index and waist circumference in midchildhood and adverse cardiovascular disease risk clustering in adolescence. Am J Clin Nutr. 2007;86:549–555. 17823416 10.1093/ajcn/86.3.549

[16] Blüher S, Molz E, Wiegand S, Otto KP, Sergeyev E, Tuschy S, et al Adiposity Patients Registry Initiative and German Competence Net Obesity. Body mass index, waist circumference, and waist-to height ratio as predictors of cardiometabolic risk in childhood obesity depending on pubertal development. J Clin Endocrinol Metab. 2013; 98:3384–3393. doi: 10.1210/jc.2013-1389 23775352 10.1210/jc.2013-1389

[17] Krakauer NY, Krakauer JC. A new body shape index predicts mortality hazard independently of body mass index. PLoS One. 2012;7:e39504 doi: 10.1371/journal.pone.0039504 22815707 10.1371/journal.pone.0039504 PMC3399847

[18] Krakauer NY, Krakauer JC. An Anthropometric Risk Index based on combining height, weight, waist, and hip measurements. J Obes. 2016;2016:8094275 doi: 10.1155/2016/8094275 27830087 10.1155/2016/8094275 PMC5088335

[19] Savva SC, Tornaritis M, Savva ME, Kourides Y, Panagi A, Silikiotou N, et al Waist circumference and waist-to-height ratio are better predictors of cardiovascular disease risk factors in children than body mass index. Int J Obes Relat Metab Disord. 2000;24:1453–1458. 11126342 10.1038/sj.ijo.0801401

[20] Sijtsma A, Bocca G, L'abée C, Liem ET, Sauer PJ, Corpeleijn E. Waist-to height ratio, waist circumference and BMI as indicators of percentage fat mass and cardiometabolic risk factors in children aged 3–7 years. Clin Nutr. 2014;33:311–315. doi: 10.1016/j.clnu.2013.05.010 23768783 10.1016/j.clnu.2013.05.010

[21] Kahn HS, Imperatore G, Cheng YJ. A population-based comparison of BMI percentiles and waist-to-height ratio for identifying cardiovascular risk in youth. J Pediatr. 2005;146:482–488. doi: 10.1016/j.jpeds.2004.12.028 15812450 10.1016/j.jpeds.2004.12.028

[22] Bertoli S, Leone A, Krakauer NY, Bedogni G, Vanzulli A, Redaelli VI, et al (2017) Association of Body Shape Index (ABSI) with cardio-metabolic risk factors: A cross-sectional study of 6081 Caucasian adults. PLoS ONE 12(9): e0185013 doi: 10.1371/journal.pone.0185013 28945809 10.1371/journal.pone.0185013 PMC5612697

[23] Tanner JM, Whitehouse RH. Clinical longitudinal standards for height, weight, height velocity, weight velocity, and stages of puberty Arch Dis Child. 1976; 51: 170–179. 952550 10.1136/adc.51.3.170 PMC1545912

[24] Flynn JT, Kaelber DC, Baker-Smith CM, Blowey D, Carroll AE, Daniels SR et al Clinical Practice Guideline for Screening and Management of High Blood Pressure in Children and Adolescents. Pediatrics. 2017;140(3):e20171904) doi: 10.1542/peds.2017-1904 28827377 10.1542/peds.2017-1904

[25] Kêkê LM, Samouda H, Jacobs J, di Pompeo C, Lemdani M, Hubert H, et al Body mass index and childhood obesity classification systems: A comparison of the French, International Obesity Task Force (IOTF) and World Health Organization (WHO) references. Rev Epidemiol Sante Publique. 2015 6;63(3):173–82. 21]. doi: 10.1016/j.respe.2014.11.003 26002984 10.1016/j.respe.2014.11.003

[26] World Health Organization. Physical status: the use and interpretation of anthropometry. Technical Report Series 854. 1995. 8594834

[27] Matthews DR, Hosker JP, Rudenski AS, Naylor BA, Teacher DF, Turner RC. Homeostasis model assessment: insulin resistance and beta cell function from fasting plasma glucose and insulin concentration in man. Diabetologia. 1985;28:412–419. 3899825 10.1007/BF00280883

[28] D'Annunzio G, Vanelli M, Pistorio A, Minuto N, Bergamino L, Iafusco D, et al Insulin resistance and secretion indexes in healthy Italian children and adolescents: a multicentre study. Acta Biomed. 2009;80:21–28 19705616

[29] Expert Panel on Integrated Guidelines for Cardiovascular Health and Risk Reduction in Children and Adolescents, National Heart, Lung, and Blood Institute. Expert panel on integrated guidelines for cardiovascular health and risk reduction in children and adolescents: summary report. Pediatrics. 2011; 128: Suppl 5: S213–S256. 22084329 10.1542/peds.2009-2107C PMC4536582

[30] Manco M, Grugni G, Di Pietro M, Balsamo A, Di Candia S, Morino GS et al Triglycerides-to-HDL cholesterol ratio as screening tool for impaired glucose tolerance in obese children and adolescents. Acta Diabetol. 2016;53:493–8 10.1007/s00592-015-0824-y 26687197

[31] Di Bonito P, Valerio G, Grugni G, Licenziati MR, Maffeis C, Manco M et al Comparison of non-HDL-cholesterol versus triglycerides-to-HDL-cholesterol ratio in relation to cardiometabolic risk factors and preclinical organ damage in overweight/obese children: the CARITALY study. Nutr Metab Cardiovasc Dis.2015;25:489–94 doi: 10.1016/j.numecd.2015.01.012 25813687 10.1016/j.numecd.2015.01.012

[32] American Diabetes Association. Classification and Diagnosis of Diabetes. Diabetes Care 2015; 38 (Supplement 1): S8–S16. 10.2337/dc15-S005 25537714

[33] Shannon A, Alkhouri N, Carter-Kent C, Monti L, Devito R, Lopez R, et al Ultrasonographic quantitative estimation of hepatic steatosis in children with NAFLD. J Pediatr Gastroenterol Nutr. 2011;53:190–195. https://doi.org/10.2337/dc15-S005 21788761 10.1097/MPG.0b013e31821b4b61 PMC3144505

[34] Nunez C, Rubiano F, Horlick M, Thornton J, Heymsfield SB. Application of Leg-to-Leg Bioimpedance System in Children. Atlanta, Ga: International Life Sciences Institute Center for Health Promotion; 1999.

[35] Zimmet P, Alberti G, Kaufman F, Tajima N, Silink M, Arslanian S, et al, IDF Consensus Group. The metabolic syndrome in children and adolescents—an IDF consensus report. Pediatr Diabetes. 2007;8:299–306. doi: 10.1111/j.1399-5448.2007.00271.x 17850473 10.1111/j.1399-5448.2007.00271.x

[36] Després JP1, Lemieux I, Bergeron J, Pibarot P, Mathieu P, Larose E, et al Abdominal obesity and the metabolic syndrome: contribution to global cardiometabolic risk. Arterioscler Thromb Vasc Biol. 2008;28:1039–1049. doi: 10.1161/ATVBAHA.107.159228 18356555 10.1161/ATVBAHA.107.159228

[37] Arner E, Westermark PO, Spalding KL, Britton T, Ryden M, Frisen J, et al Adipocyte turnover: relevance to human adipose tissue morphology. Diabetes. 2010;59:105–109. doi: 10.2337/db09-0942 19846802 10.2337/db09-0942 PMC2797910

[38] Kelishadi R, Keikha M. Systematic review on the association of abdominal obesity in children and adolescents with cardio-metabolic risk factors. J Res Med Sci. 2015; 20: 294–307. 26109978 PMC4468236

[39] Maggio AB, Mueller P, Wacker J, Viallon M, Belli DC, Beghetti M, et al Increased pancreatic fat fraction is present in obese adolescents with metabolic syndrome. J Pediatr Gastroenterol Nutr. 2012;54:720–726. doi: 10.1097/MPG.0b013e318244a685 22157928 10.1097/MPG.0b013e318244a685

[40] Toledo-Corral CM, Alderete TL, Hu HH, Nayak K, Esplana S, Liu T, et al Ectopic fat deposition in prediabetic overweight and obese minority adolescents. J Clin Endocrinol Metab. 2013;98:1115–1121. doi: 10.1210/jc.2012-3806 23386647 10.1210/jc.2012-3806 PMC3590481

[41] Giorgio V, Prono F, Graziano F, Nobili V. Pediatric nonalcoholic fatty liver disease: old and new concept on development, progression, metabolic insight and potential treatment targets. BMC Pediatr. 2013; 13:40 doi: 10.1186/1471-2431-13-40 23530957 10.1186/1471-2431-13-40 PMC3620555

